# Veterinary Medical Association of New Jersey

**Published:** 1885-10

**Authors:** Wm. Herbert Lowe

**Affiliations:** Secretary


VETERINARY MEDICAL ASSOCIATION OF NEW JERSEY.

The fifth regular meeting of the Veterinary Association of New Jersey was held at Van Woert's Hotel, Long Branch, August 6th, 1885.

The President, Dr. Miller, called the meeting to order at 1.30 P.M., and requested the Secretary to call the roll. The following members were in attendance: Drs. H. W. Rowland, of Jersey City; Wm. B. E. Miller, of Camden; C. K. Dyer, of Mount Holly; James W. Hawk, of Newark; W. P. Humphreys, of Elizabeth; Wm. B. Haydon, of Newark; J. C. Dustan, of Morristown; J. Gerth, Jr., of Newark; and Wm. Herbert Lowe, of Paterson.

The minutes of the last meeting were read and approved.

Dr. W. H. Purdy, Secretary of the New York State Veterinary Society, was present. Dr. W. Runge, of Newark, was also present as a visitor. The secretary received a letter from Prof. Liautard, Dean of the American Veterinary College, expressing his desire and intention to be present at the meeting; but his absence was accounted for by the pressure of duties which necessarily demanded his time on his return from his recent sojourn in Europe. A telegram was received from Dr. Chas. T. Goentner, Secretary of the Keystone Veterinary Medical Association, expressing his inability to attend.

According to Senator Fish's advice and instructions, the Certificate of Incorporation had been duly recorded at Trenton. Therefore, the Veterinary Medical Association of New Jersey has become legally incorporated.

The chair requested the committee upon the Constitution and By-Laws to report. Dr. Lowe, on behalf of the committee, stated that the new Constitution and By-Laws were in course of preparation, but as Dr. Gerth had been traveling in the West, it had been impossible for them to meet in time to complete the same.

The reports of the Secretary and Treasurer were received.

Dr. Dustan related, at some length, his experience with rheumatic influenza, stating that twenty-two cases had come under his observation during the present year. Considerable discussion followed Dr. Dustan's statements, which were participated in by many of the members. Dr. Lowe spoke of two cases of so-called rheumatic influenza, and gave some points of differentiation.

Dr. Dyer was the next speaker, the subject being operative surgery, and gave his experience in removing melanotic tumors. Dr. Miller followed on the same subject.

Dr. Rowland, who has been making investigations for the government in Texas relative to contagious diseases, was asked to give an account of his investigations, but declined to do so at the present meeting.

The Secretary offered the following resignation;

"LONG BRANCH, N. J.,
August 6th, 1885.

*To the Veterinary Medical Association of New Jersey.*

GENTLEMEN—The undersigned, your secretary, most respectfully tenders his resignation as Secretary of the Veterinary Medical Association of New Jersey. This resignation is a necessity, owing to the fact that I leave New Jersey permanently to make a new home in the State of Nebraska. I exceedingly regret taking this step, but assure your honorable body that my association with your society will always be remembered with pleasure and pride.

Yours most truly,
J. GERTH, Jr."

The resination was accepted. The President, Dr. Miller, said that next in order would be the election of a Secretary. Dr. Lowe was nominated and elected unanimously.

The following resolutions were offered by the President:

"WHEREAS, our Secretary, Dr. Julius Gerth, Jr., has recently been appointed State Veterinary Surgeon of Nebraska, and, consequently, is obliged to leave us; therefore, be it

"*Resolved*, that we express our sorrow at the loss of so valuable a member of this Association, but at the same time we congratulate ourselves upon the appointment of one of our members to so responsible a position in a far-distant State, and we feel assured that the authorities of the State of Nebraska will have no cause to regret their choice;

"*Resolved*, that we tender to our esteemed friend our hearty congratulations, and hope his appointment may prove equally beneficial to both the State and the appointee;

"*Resolved*, that we express the hope that the health of our friend may be preserved to a long life of usefulness and influence in his future field of operations."

Dr. Gerth's response, upon the adoption of the resolutions, was characterized by marked appreciation of the esteem in which he was held by his brother members of the Association, and he then offered his resignation as a member of the Society. The same was accepted, and he was immediately and unanimously elected an Honorary Member.

The Secretary was instructed to forward a copy of the foregoing resolutions to his Excellency, Hon. James W. Dawes, Governor of Nebraska.

It was decided to hold the next regular meeting in Camden, the second Thursday in December.

WM. HERBERT LOWE, D.V.S., Secretary.

